# Educational Needs Assessment of General Practitioners in Tuberculosis Control and Management

**Published:** 2019-03

**Authors:** Somaye Sohrabi, Yalda Soleiman Ekhtiari, Sareh Shakerian

**Affiliations:** 1 School of Management and Medical Education, Shahid Beheshti University of Medical Sciences, Tehran, Iran,; 2 Social Determinants of Health Research Center, Shahid Beheshti University of Medical Sciences, Tehran, Iran,; 3 Departments of Community Based Education of Health Sciences, School of Management and Medical Education, Shahid Beheshti University of Medical Sciences, Tehran, Iran.

**Keywords:** Educational needs, General practitioner, Tuberculosis

## Abstract

**Background:**

Tuberculosis is one of the top 10 causes of mortality worldwide. It is also the leading cause of death in HIV-positive patients. In this study, we aimed to assess the educational needs of general practitioners regarding tuberculosis in the North Health Center of Tehran, Iran.

**Materials and Methods:**

This quantitative and qualitative study was conducted in 2017. In the quantitative phase, 31 general practitioners from the North Health Center of Tehran were included. The educational needs assessment was performed using the knowledge assessment and self-assessment scales. Data were entered in SPSS version 21 and analyzed using descriptive tests and Pearson’s correlation coefficient test. In the qualitative phase, data were collected by interviewing six managers of tuberculosis monitoring program and analyzed using the content analysis method.

**Results:**

The mean score of the knowledge assessment scale was 22.8±6.4. The most and the least important educational needs were related to treatment and general information about tuberculosis, respectively. Moreover, tuberculosis treatment and general information about tuberculosis were the most and the least important educational needs in the self-assessment scale, respectively. There was a poor correlation between the mean scores of self-assessment scale and knowledge assessment scale regarding tuberculosis prevention (P=0.01, r=0.27). Also, a moderate correlation was found regarding tuberculosis screening (P=0.001, r=0.56). However, no significant correlation was found in terms of general information (P=0.31), diagnosis (P=0.43), and treatment (P=0.29) of tuberculosis. Five major themes were extracted in the qualitative phase of the study, including “training time”, “educational content”, “educational references”, “teaching method”, and “organizational factors”.

**Conclusion:**

An appropriate educational program should be developed for general practitioners in form of continuing education and educational reform.

## INTRODUCTION

Tuberculosis (TB) is a global health problem and one of the top 10 causes of mortality worldwide. Evidence suggests that TB is responsible for more deaths than HIV and malaria. An estimated number of 10.4 million new incidents of TB were reported in 2015, and about 1.8 million people died from TB worldwide (1). Every second, a person is infected with TB around the world, and every 10 seconds, a person dies from TB. A sputum-positive person can infect 10–15 people in a year. Therefore, early detection of TB cases and prompt treatment are imperative for successful TB control in the community (2).

Globally, the number of TB deaths fell by 22% from 2000 to 2015 (1). Pervasive poverty, lack of proper management, HIV/AIDS epidemic, poor public health services, rapid population growth, and rapid urbanization are contributing factors for the re-emergence of TB (3). On the other hand, inadequate or inappropriate treatment and non-compliance with the National Tuberculosis Control Program (NTCP) guidelines have influenced the emergence of refractory drug-resistant forms of TB (4).

According to the latest statistics published by the Ministry of Health and Medical Education of Iran in 2015, the number of pulmonary TB cases with positive sputum smears was 6.26 per 100,000 people in Iran, with the highest incidence and prevalence rates reported in Sistan and Baluchistan and Golestan provinces, respectively (5). India, Indonesia, China, Nigeria, Pakistan, and South Africa are six countries, which account for 60% of new cases of TB (1). Statistics show that the rate of TB and drug-resistant TB in neighboring countries of Iran, such as Pakis tan (6), Afghanistan (7), and Iraq (8), is several times higher than Iran.

The fight against TB continues around the world. Despite significant progress in the 19^th^ and 20^th^ centuries, TB is still not eradicated in many countries, especially in developing countries (9). Success of the Direct Observed Treatment, Short Course (DOTS) rests on the healthcare system’s ability to identify and follow-up suspected TB cases. Delay in diagnosis or initiation of effective treatment leads to the transmission of this infection to susceptible contacts in the community, increases patient expenditure, overburdens the health system, and increases the risk of mortality (2).

In Iran, general practitioners (GPs) working in health centers provide major care for patients with TB under the supervision of infectious diseases specialists, while private-sector physicians are responsible for part of the care. Adequate knowledge and suitable functioning of physicians are essential to the success of TB control programs (10). Needs assessment is one of the most controversial concepts in the field of education, training, and curriculum planning (11). It is an essential component of the educational planning process, formulation of plans, and adoption of educational measures (12).

Grant believes that if education, particularly medical education, is based on the needs assessment and function of individuals, it can change their behaviors (13). The results of various studies indicate that physicians’ knowledge of TB diagnosis, transmission, and treatment is inadequate (10, 14). Therefore, in this study, we aimed to assess the educational needs of GPs in the North Health Center of Tehran, Iran about NTCP. We also aimed to determine the viewpoints of middle and senior managers regarding the implementation of NTCP.

## MATERIALS AND METHODS

### Study Design and Population

This study was conducted in two quantitative and qualitative phases. The quantitative phase was implemented in the North Health Center of Tehran, and the qualitative phase was implemented in the monitoring units for NTCP in Tehran. The study was performed from March 2017 until November 2017. Due to the limited number of GPs working in Tehran North Health Center, all of GPs (n=31) were selected via census sampling and included in the quantitative phase.

The TB experts of Tehran North Health Center, infectious diseases and TB experts of the Health Department of Shahid Beheshti Medical University (SBMU), two experts from the Department of Tuberculosis and Leprosy of the Ministry of Health and Medical Education of Iran, and one community medicine specialist from SBMU were the middle and senior managers, who worked in TB control and monitoring units. The experts were selected via purposive sampling method.

### Implementation and data collection

In the quantitative phase, after coordination with the manager of the North Health Center of Tehran, the researcher attended physician retraining sessions for two days and had access to all physicians. The participants completed the knowledge assessment and self-assessment questionnaires. In the qualitative phase, appointments were scheduled after contacting the managers via phone calls and giving them explanations about the study. The researcher met the experts in the designated place at the designated time and explained the purpose and methods of the study.

The interviews were conducted in a quiet place in accordance with the principles of communication. Each interview lasted between 45 and 60 minutes. At the end of each interview, we thanked the participants and allowed them to discuss any specific points or items. Recruitment was stopped when saturation was reached for the key themes. The interviews were recorded after obtaining the participants’ permission. We listened to the recordings immediately after the interviews and transcribed them word-by-word. The interviews were then reviewed several times for immersion, and the themes were extracted.

### Questionnaires

The knowledge assessment questionnaire was based on an assessment tool by Mohammadi which included demographic information and 40 questions about the knowledge of TB (15). The knowledge questions were about the general knowledge of TB (n=5), prevention (n=9), screening (n=5), diagnosis (n=8), and treatment (n=13). It was a multiple-choice questionnaire. On the other hand, the self-assessment questionnaire (16) consisted of 22 items rated on a three-point Likert scale (“retraining is required”, “retraining and updating of content are required”, and “education is not necessary”). The questionnaire was organized in five parts of general knowledge about TB (n=10), prevention (n=2), screening (n=3), diagnosis (n=2), and treatment (n=5). Cronbach’s alpha was also calculated to be 0.958 for the knowledge assessment scale and 0.862 for the self-assessment questionnaire.

A semi-structured interview was conducted in the qualitative phase of the study. Two main questions were asked in the interviews:

- What are the required competencies for GPs in order to manage and control TB?
- What is the most important education requirement for GPs to manage and control TB?

Also, the participants were asked to state their comments and suggestions to improve the current status of TB control and management. The validity and reliability of the qualitative phase were different from the quantitative phase.

### Ethical considerations

The study protocol conformed to the ethical guidelines of the Declaration of Helsinki. The Ethics Committee of SBMU approved the study (ethics code: IR.SBMU.RETECH.REC.100/808). Before the study, the researcher explained the importance of study implementation. Participation in the study was completely voluntary, and the questionnaires remained anonymous. A written informed consent was also obtained from each participant.

### Statistical analysis

In the quantitative phase, data were imported into SPSS version 21 (IBM Corp., Chicago, IL, USA) and presented as mean±SD and frequency (%). Descriptive methods and Pearson’s correlation coefficient test were used to analyze the findings. In the qualitative phase, data collection and analysis were performed simultaneously, and data were analyzed via qualitative content analysis. After full immersion in the context of the interviews, data were broken into smaller meaningful chunks, and categories and subcategories were extracted. During the sessions, the process of theme extraction was reviewed by the supervisor.

## RESULTS

### Quantitative phase

Of 31 GPs, 7 (22.6%) were male, and 24 (77.4%) were female. The mean±SD of the participants’ age was 46.5±5.2 years, and the mean±SD of work experience was 18.06±5.6 years. Table 1 shows the participants’ knowledge of NTCP. The lowest score of the knowledge test was four, and the highest score was 38 out of 40, with the mean±SD of 22.8±6.4. The scores of the questionnaires were classified as weak (<40%), moderate (40–60%), and good (>60%). More than half of the participants could not correctly answer the questions about TB treatment (55.8%) and had poor knowledge in this area. Their level of knowledge was moderate (50%) for the diagnosis of TB, while they had good knowledge about the general information of TB (79.3%), TB prevention (64%), and TB screening (67.7%). Table 2 presents the results of participants’ self-assessment about NTCP. Most of the participants stated that they needed retraining about TB treatment (83.2%) and TB diagnosis (85.5%). The need for training was high in other areas, but it was less than the mentioned areas.

**Table 1.** Participants answer to knowledge assessment questions

**Fields**	**Correct answer**	**Wrong answer**	**I don’t know**
	**No.(%)**	**No.(%)**	**No.(%)**
General information about (questions=5)	123 (79.3)	24 (15.5) TB	8 (5.2)
Prevention (questions =9)	176 (64.0)	80 (27.7)	23 (8.2)
Screening (questions =5)	105 (67.7)	33 (21.3)	17 (11.0)
Diagnosis (questions =8)	124 (50.0)	86 (34.7)	38 (15.3)
Treatment (questions =13)	178 (44.2)	137 (34.0)	88 (21.8)
Sum (questions =40)	706 (57.0)	360 (29.0)	174 (14.0)

**Table 2.** Self-assessment results

**Fields**	**Retraining is required No.(%)**	**Retraining and updating of content is required No.(%)**	**Education is not necessary No.(%)**
**General information about TB (questions =10)**	71 (22.9)	108 (34.8)	131 (42.3)
**Prevention (questions =2)**	16 (25.8)	18 (29.0)	28 (45.2)
**Screening (questions =3)**	26 (28.0)	44 (47.3)	23 (24.7)
**Diagnose (questions =2)**	22 (35.5)	31 (50.0)	9 (14.5)
**Treatment (questions =5)**	55 (35.5)	74 (47.7)	26 (16.8)

Pearson’s correlation coefficient test was used to analyze the data. The results indicated that there was no significant correlation between the mean self-assessment scores and the mean scores of knowledge test regarding general information about TB (P=0.31). There was a weak correlation between the mean scores of self-assessment and the mean scores of knowledge test in the area of TB prevention (P=0.01, r=0.27). On the other hand, a moderate correlation was observed between the mean self-assessment scores of TB screening and the mean scores of knowledge test in this area (P=0.001, r=0.56). However, there was no significant correlation between the mean self-assessment scores of TB diagnosis and the mean scores of knowledge test in this area (P=0.43). Similarly, there was no significant correlation between the mean self-assessment scores of TB treatment and the mean scores of knowledge test in this area (P=0.29).

### Qualitative phase

In the qualitative phase of the study, a total of 300 semantic units were extracted. After reviewing the units which were similar to each other, they were merged, and nearly 180 initial codes were extracted. The primary codes were classified into categories and subcategories. Finally, five main themes emerged by interviewing the managers: “training time”, “educational content”, “educational references”, “teaching method”, and “organizational factors” (Table 3).

**Table 3.** Themes, Categories and sub categories

**Theme**	**Categories**	**Sub categories**
Training time	Student period	- Theoretical education
		- Practical education
	Work period	- In-service training
Educational content	Nature of the disease and its management	- principles and generalities
		- pathology and physiopathology,
		- Screening
		- Diagnosis
		- Treatment
		- Prevention
	social aspects of the disease	- Patient in the family
		- Patient in the community
	Teaching methods to patient and his/ her family	- Face to face training
		- written (pamphlet or a booklet) training
	Correct communication skills	- proper communication with patient
		- proper communication with patient's family
		- psychological topics
	Patient workflow in health center	- Screening
		- Diagnosis
		- Following up
Educational references	Reference books	- reference books like Harrison
Teaching method	Theoretically	- DVD
		- Pamphlet
		- Classroom
	Practically	- In health centers with TB affected patient
		- In prisons
		- In addiction treatment centers
Organizational factors	Motivation	- Assign organizational position for physicians
		- Reducing their workload
	Financial issues	- Establishing a fair payment system for physicians
		- Financial incentives
		- Incentive pay for each new detection and final treatment

### Training time

This theme consisted of two categories of students and work period. In this regard, the managers stated:

“*In two stages of medical education, students are involved in health education courses and health activity assessment in health centers. In these stages, it’s possible to learn the theory of diseases in parallel with practical training, focusing on infectious diseases, which are prioritized at the ministry level.”*

“Physicians working in healthcare centers should have theoretical and practical training.”

### Educational content

This theme included the nature of the disease, disease management, social aspects of the disease, patient/family education, communication skills, and workflow related to TB in health centers. The participants mentioned the need for learning the nature of the disease and its management. Some of the participants remarked:

“A medical student should understand the principles and generalities of tuberculosis, global policies of tuberculosis control, diagnostic methods of pulmonary tuberculosis, and DOTS.” “Physicians should be able to treat certain conditions, such as tuberculosis during pregnancy and breastfeeding or tuberculosis in patients with liver and kidney diseases. It is important for physicians to be familiar with the side effects of drugs and drug resistance, management and monitoring of the disease during treatment, contact management, tuberculosis in children, tuberculosis prevention, tuberculosis prevention in infants of mothers with tuberculosis, and tuberculosis control in prison.”

### Educational references

This theme consisted of two categories of reference books and instructions. Guidelines can be regional, global, or issued by the Ministry of Health. In this regard, some of the participants said:

“Effective learning of reference books, such as Harrison’s principles, is very important during training.”

“Physicians should be familiar with the national, regional, and global guidelines in healthcare centers to learn how to deal with a TB suspect or afflicted person.”

Regarding the location of training, three categories were extracted: university classes, health centers, and others (prisons or rehabilitation centers). In this regard, one of the participants remarked:

“The course content related to infectious diseases should be specifically designed to make students fully familiar with the theories at university”.

### Teaching method

The next theme extracted from the interviews was the “teaching method”, which was divided into two categories: theoretical and practical training. The theoretical method was applied in classrooms, using DVDs and pamphlets, while the practical method involved teaching in health centers or training in prisons and rehabilitation centers. Some of the participants said:

“Training in centers where a patient with tuberculosis is being treated can be useful for a medical practitioner who has just started medical practice.”

“It should be noted that HIV-positive prisoners are very vulnerable to tuberculosis. We should not overlook the practical training of physicians in these centers.”

### Organizational factors

The last extracted theme was “organizational factors”. This theme consisted of organizational position and financial issues. From the participants’ viewpoint, organizational factors are important factors in improving the performance of physicians:

“The high workload, low salary, and lack of payment to health center physicians do not motivate them enough to work efficiently.”

“Lack of organizational positions for TB coordinators, unfair payment systems for physicians working in health centers, lack of organizational charts, and problem of scarce manpower for active screening in health centers are factors that reduce the motivation of physicians.”

## DISCUSSION

Comparison of the present results showed some similarities in the quantitative and qualitative phases. The participants answered 43% of the questions incorrectly, and 71.3% of them expressed that they needed retraining in the self-assessment checklist. On the other hand, the managers emphasized the importance of theoretical and practical training of NTCP in undergraduate and in-service courses. According to the present results, there is a need for retraining NTCP, especially in the field of TB treatment, prevention, and diagnosis.

Based on the interviews with the managers, some issues encouraged the researcher to conduct further explorations in the future. For example, attention to individual factors is one of the main factors for improving the performance of physicians. To this end, development of organizational rows for TB coordinators, reduction of workload, increase of labor force, and establishment of a fair payment system for physicians working in health centers should be considered. There were also some new points in the interviews with the managers, which were not identified in the quantitative phase, including communication with the patients and their families, training of patients and their families, and discussions about psychological, individual, and social issues.

Similar studies in this area confirm the results of the present study. In a study conducted in Ethiopia, the knowledge and practice of private-sector physicians were assessed in the control of TB based on DOTS strategy. It was found that appropriate care and treatment were only provided by 50% of the physicians. Overall, 39.31% of the physicians did not have sufficient knowledge about DOTS (17). Poor knowledge of physicians about NTCP and need for training courses have been also emphasized in other studies (18). Naseer et al. showed a significant difference in the level of knowledge between physicians who participated in the NTCP training course and those who did not attend the course; therefore, the need for training courses was highlighted (19).

Moreover, Hoffman and colleagues showed that it is necessary to consider the role of education and attract the attention of policymakers and international institutions to strategies needed to improve the knowledge and practice of physicians about TB (20). Mombini et al. found that a major factor, which causes physicians not to use their knowledge in practice, is inadequate education about TB in universities. This shortcoming was evident in some of the key aspects of TB approaches (21). In this context, various studies have focused on teaching NTCP to medical students, as well as the rotation of students in clinics with DOTS-related activities (3). Moreover, effective measures must be taken to increase the family physicians’ knowledge of TB, and TB dispensary workers must have the same economic status as family physicians (14).

According to the results of the present study, it is suggested that medical students learn the educational requirements of their future profession and apply their knowledge in their professional practice in a continuous and effective manner. It is also necessary to design an educational curriculum based on the needs of GPs for their professional practice in TB care systems, implement postgraduate continuing education programs, and integrate postgraduate monitoring systems to improve their professional performance. Clinical students should also work in TB clinics next to experienced physicians for at least two weeks. Finally, it is recommended that health authorities reduce the workload of GPs, assign them to high organizational positions, and increase their salary in order to motivate them and improve their performance.

## CONCLUSION

Based on the results of the present study, medical education about TB is relatively poor for GPs working in the North health Center of Tehran. Therefore, an appropriate educational program should be developed for GPs about NTCP in form of continuing educational programs and educational reforms.
